# Supplementary material for: Acute influence of an adaptive sporting event on quality of life in veterans with disabilities
Source: PLoS One. 2022 Nov 28;17(11):e0277909. doi: 10.1371/journal.pone.0277909 (PMC9704555; doi:10.1371/journal.pone.0277909)
Supplement: Attachment — Submitted filename: Letter of Response.pdf [file pone.0277909.s001.pdf]

September 29, 2022
Dr. Yih-Kuen Jan
Academic Editor
PLOS ONE

Subject: Revision and resubmission of manuscript PONE-D-22-10455

Dear Dr. Yih-Kuen Jan,

Thank you for your email and the opportunity to revise our manuscript entitled “Acute Influence of an Adaptive Sporting Event on Quality of Life in Veterans with Disabilities”. We greatly appreciate the reviewers for their insightful comments. The manuscript has been improved after making the suggested edits.

Please find attached a point-by-point response to the reviewer’s concerns. The revision has been developed in consultation with all coauthors. We hope that you find our responses satisfactory, and that the manuscript is now acceptable for publication. We thank you for your continued interest in our research.

Sincerely,

Alexis Sidiropoulos, PhD
Department of Veterans Affairs
New York Harbor Healthcare System

Journal Requirements Addressed:

Comment 1: PLOS ONE style requirements for file naming.

Response: Following the guidance, we have created separate files for manuscript figures with appropriate file types and names.

Comment 2: Statement of where the minimal data set underlying the results described in the manuscript can be found.

Response: We have included information in the Data Availability Statement indicating that the minimal data set underlying the results described in this manuscript can be found in Dryad Digital Repository. Dryad is a curated resource that makes the data underlying scientific publications discoverable, freely usable, and citable while promoting openly available research integrated with scholarly literature.

Comment 3: Please include your full ethics statement in the 'Methods' section of your manuscript file. In your statement, please include the full name of the IRB or ethics committee who approved or waived your study, as well as whether or not you obtained informed written or verbal consent. If consent was waived for your study, please include this information in your statement as well.

Response: We have included additional statements within the 'Methods' section to clearly state that the Department of Veteran Affairs New York Harbor Healthcare System Institutional Review Board waived the consent of participants for this study due to its retrospective nature.

Reviewer 1:

Comment 1: Only 58 of those participants completed both the pre- and post-survey, 27% participants refused to answer the survey, please explain the reason.

Response: We thank the reviewer for their note about the percentage of event participants that did not complete the survey. We would like to note that none of the participants refused, however there were many reasons why these participants did not complete the survey. Some examples include beginning the event without having completed the pre-survey and leaving the event without knowing that post-survey information was requested.

Comment 2: Veterans who participated in only a one-day kayaking and sailing event, time is too short. How to figure out the other reasons may lead to the differences about quality of life.

Response: In agreement with the reviewer, we list this short time period as a limitation in our study. Further, we suggest that future research should include a follow-up survey to determine if the positive acute effects on quality of life are retained long-term. Please see the "Limitation and future research" section.

Comment 3: The Influence on Quality of Life Scale construct validity should be reported. Quality of life for athletes with cerebral palsy may do not suit for veterans with disabilities.

Response: In agreement with the reviewer, statistical analyses were conducted to confirm the validity and reliability of the survey results. This information is included an additional sub-section within the "Results" termed "Validity and Reliability".

Comment 4: In the analysis, the manuscript should add age, sex and other variable as confounder variable in the analysis.

Response: Additional statistical analyses have been conducted to identify any confounding participant characteristics. Further, a regression model fit to the data determined the contribution of each participant characteristic to the survey sum score. The additional statistical analyses are reported in the second paragraph of the "Statistical Analysis" sub-section of the "Methods" section. Please see the sub-sections under the "Results" sections entitled "Participant Characteristics" and "Sum Score Analysis".

Reviewer 2:

Comment 1: In Line 54-63 (INTRODUCTION), it seems better to pinpoint the "gap", the research gap which previous studies have left but your research will fill. For example, your research highlights the ACUTE influence of participation in A SINGLE-DAY, so is this an aspect that previous studies ignore but your research notice? If so, please elaborate on this "gap", which seems one of your novelty worth highlighting.

Response: We thank the reviewer for this comment and have added information into the introduction to address the gap in the literature which this research fills. Please see lines 59-65.

Comment 2: In DISCUSSION, to corroborate the current study, the authors EXTENSIVELY discuss the previous research (esp. on whether the adaptive sports improve the mental health & QoL of the disabled), but it seems better to add extra information about the current study's RESULTS, among which several interesting details seem worth further discussing/explaining:

2.1: As the authors mentioned in Line 148 & Table 2, after participation, the OEF/OIF veterans had the greatest positive change scores, compared with veterans during other military service eras. This data is so striking that it seems better to briefly explain the possible reasons and implications for this trend.

2.2: In Table 2, after participation, a decrease of QoL/QoSL was reported by veterans with orthopedic or spinal cord injury. This data is eye-catching and worth a brief discussion/explanation.

Response: Following the updated statistical analyses, previously discussed trends were no longer valid. However, we have included an updated discussion section from lines 196-217 to discuss the adjusted findings.

Comment 3: In REFERENCES, over 91% (43/47) of references have been published for over 5 years, thus seeming "old". It seems better to cite more RECENT references (published after 2016). Since 2016, there have been at least 60 papers in the field of "(adaptive sport*") AND disab*, such as PMID: 31046012, PMID: 32427531, and PMID: 3247531.

Response: In agreement with the reviewer, we have updated 11 citations in the "References" section with more recent publications. However, due to the limited research published in Veteran- and amputee-specific adaptive sports programs, some "older" references remain in the manuscript. We believe this highlights the need for further research in this field.
